# Network Analysis of the Relationship Between Trait Depression and Impulsiveness Among Youth

**DOI:** 10.3389/fpsyt.2022.916332

**Published:** 2022-06-17

**Authors:** Jingxuan Zhang, Kuiliang Li, Yitong Xue, Zhengzhi Feng

**Affiliations:** Department of Medical Psychology, Army Medical University, Chongqing, China

**Keywords:** trait depression, impulsiveness, network analysis, facet, dysthymia, anhedonia, youth

## Abstract

**Objective:**

Both impulsiveness and trait depression are the trait-level risk factors for depressive symptoms. However, the two traits overlap and do not affect depressive symptoms independently. This study takes impulsiveness and trait depression into a whole construct, aiming to find the complex associations among all facets and explore their relative importance in a trait network. It can help us find the key facets that need consideration in preventing depression.

**Materials and Methods:**

We used the Barratt Impulsiveness Scale (BIS) and Trait Depression Scale (T-DEP) as measuring tools, conducted network analysis, and applied the Graphic Least Absolute Shrinkage and Selection Operator (GLASSO) algorithm to estimate the network structure and compute the linkage and centrality indexes. The accuracy and stability of the indexes were estimated through bootstrapping. All the computations were performed by R script and packages.

**Results:**

We found that “trait anhedonia” was connected with “non-planning” and “cognitive” impulsiveness, while “trait dysthymia” was connected with “motor” impulsiveness. “Cognitive” impulsiveness had a statistically significant higher expected influence than “motor” impulsiveness and had the trend to be dominant in the network. “Trait dysthymia” had a statistically significant higher bridge expected influence than “cognitive” impulsiveness and had the trend to be the key facet linking impulsiveness with trait depression. “Non-only children” had higher network global strength than “only children.” All indexes were accurate and stable.

**Conclusion:**

The present study confirms the complex associations among facets of trait depression and impulsiveness, finding that “cognitive” impulsiveness and “trait dysthymia” are the two key factors in the network. The results imply that different facets of impulsiveness should be considered respectively regarding anhedonia and dysthymia. “Cognitive” impulsiveness and “trait dysthymia” are critical to the prevention of depression.

## Introduction

Impulsiveness is an important risk factor for several mental disorders, including depression (1–3). The causal effect of impulsiveness on depression is confirmed by a longitudinal study (3). This indicates that impulsiveness is a vulnerability to depression. However, as assessed by the big-five personality inventory, trait depression predicts depressive symptoms, while impulsiveness does not (4). Trait depression and impulsiveness are two correlated facets of the domain “Neuroticism” (5), which is also verified when the two traits are measured by independent scales (6). Consequently, we infer that impulsiveness does not predict depressive symptoms independently but acts as an overlapped vulnerability with trait depression. Therefore, it is necessary to put the two traits together rather than to view them as independent factors of depression. However, both impulsiveness and trait depression are complex concepts, including distinct facets. It is not clear enough which facets are dominant or how the two traits are correlated with each other on the facet level. This is critical to understand the trait basis of depression and to take proper measures for prevention.

There are different models of impulsiveness. In the neurocognitive domain, Fineberg et al. (7, 8) divided impulsiveness into the motor, disadvantageous decision-making, choice, and reflection. Barratt’s model, in turn, regards impulsiveness as a trait and also includes neurocognitive-related components, which are widely used in studies. It divides impulsiveness into the following three facets: “non-planning,” “motor,” and “cognitive” (9) (refer to Supplementary Table 1 for facets and definitions). Both Eysenck’s personality model (10) and the big-five personality model (5) include a depressive trait component, but do not consider the core feature of depression, i.e., anhedonia. Spielberger’s model divides depression into anhedonia (lack of pleasure) and dysthymia (existence of despondent mood) (11), which can well cover the core features of depression (12). Meanwhile, Spielberger’s model includes both state and trait depression. Different from state depression which refers to depressive symptoms or emotions now, trait depression, which includes “trait anhedonia” and “trait dysthymia,” represents general depressive feelings throughout a long period of time (11, 13).

There is evidence that various facets of impulsiveness are associated with depression. The self-reported facets, such as “urgency” (14–16), “lack of perseverance” (16), “inattention,” “lack of planning,” and “inability in controlling temper or behavior” (17), are positively correlated with depression. In addition, the behavioral-measured impulsive decision-making and disinhibition of response are significantly different in depressive participants compared to the control groups (2). However, the above studies do not consider trait-level depression and its facets.

Although there are few studies discussing the relationships between trait anhedonia and impulsiveness or between trait dysthymia and impulsiveness, existing evidence shows that state (or diagnosed) anhedonia and dysthymia are related to different kinds of impulsiveness. Some studies reveal that state anhedonia and impulsiveness are positively correlated (18, 19), while others find negative associations (20, 21). It may be due to the use of different concepts of impulsiveness [e.g., dysfunctional impulsiveness (19), impulsive personality (18), and delay discounting rate (20)]. The relationship between state dysthymia and impulsiveness is unknown with few studies discussing it. Nevertheless, there is evidence that state dysthymia co-occurs with borderline personality disorder (22, 23), which shows impulsive features. Above all, whether different facets of impulsiveness and trait depression are correlated is not clear.

We do not find any study discussing the relative importance of different facets of impulsiveness and trait depression. Therefore, we cannot come up with a hypothesis about which facet is dominant in the construct. However, there is indirect information about the associations between the facets of depression and impulsiveness. Reward processing impairment is one of the core features of anhedonia in depression (24). Depressive patients with anhedonia show reduced positive emotions for the future reward, which is explained as a lack of anticipatory pleasure (25). This symptom presents a kind of non-planning-for-the-future character, which is also included in Barratt’s impulsiveness model (9). Mood distress, as one of the features of dysthymia, is related to cognitive control (26) that is associated with lacking cognitive or behavioral inhibition. This indicates that dysthymia may be correlated with another two factors of Barratt’s model, namely, “motor” and “cognitive” (9).

Therefore, in the present study, we take Spielberger’s state-trait depression model (11) and Barratt’s impulsiveness model (9, 27) to explore the relative importance and complex relationship between the facets of trait depression and impulsiveness. Simultaneously, we are aiming to find the key facets that link the two traits and to explore whether the demographic variables can influence the whole correlation pattern and the total correlation strength of all the facets. Network analysis is a suitable method to cover both the analyses of intercorrelations (represented by edge weight) and relative importance (represented by centrality indexes) (28). It can also consider the linkage of one variable among two or more communities (29), compare the whole correlation patterns and the total correlation strength between different populations (30), and give clear visualized results (28). These are not what the traditional correlation analysis possesses. Therefore, we apply network analysis (28), which includes all the trait facets in a network with all facets as nodes and associations as edges, covering both the analyses of intercorrelations (represented by edge weight) and relative importance (represented by centrality indexes). According to the association pattern on the state (or diagnostic) level mentioned above, we hypothesize that “trait anhedonia” is more closely connected with “non-planning” impulsiveness, while “trait dysthymia” is more closely connected with “motor” and “cognitive” impulsiveness. Regarding the relative importance of the facets and the other study objectives, we refer to the posterior results.

## Materials and Methods

### Participants

**FIGURE 1:**
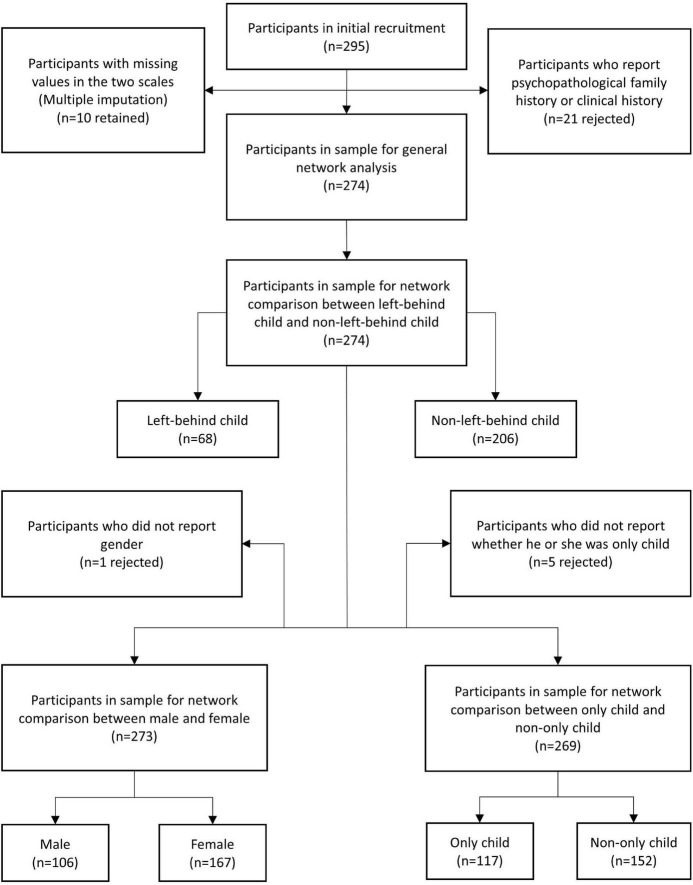
The PRISMA diagram of the participants’ recruitment process. “*n*” represented the sample size.

A total of 295 participants (female = 181, male = 113, not report gender = 1) who were under 40 years (mean = 20.71, SD = 2.97) were recruited initially. All the participants were youth studying in academies or working in Chongqing, China. They completed a paper version of the Trait Depression Scale (T-DEP) and Barratt Impulsiveness Scale (BIS) under the instruction of surveyors. We rejected 21 participants because they reported psychopathological family history or clinical history. The valid sample contained 274 participants (female = 167, male = 106, not report = 1), aging from 17 to 34 years (mean = 20.70, SD = 3.01) (refer to Table 1). There were 10 participants who had missing values in the two scales (seven participants with one missing value and three participants with two missing values each) [refer to Figure 1 for the PRISMA diagram (31) of the participants’ recruitment process]. We addressed these missing values with multiple imputation. This imputation method generates two or more values for each missing value through a certain algorithm and creates several imputation datasets. Researchers choose one or took the average of all datasets for the analysis (32). In this study, we used R “mice” package (33), applying a predictive mean matching algorithm to process multiple imputations. There were one and five participants who had missing values in gender and “only child” variables, respectively. These participants were rejected when gender or “only child” was considered in the network comparisons.

**TABLE 1:** Demographic information and descriptive statistics.

	Mean ± SD	*n*	%
Age	20.70 ± 3.01		
Gender (male)		106	38.7
Gender (female)		167	60.9
Gender (not report)		1	0.4
Only child (yes)		117	42.7
Only child (non)		152	55.5
Only child (not report)		5	1.8
Left-behind child (yes)		68	24.8
Left-behind child (non)		206	75.2
BIS	73.12 ± 13.91		
Non.I	22.51 ± 6.03		
Mot.I	26.95 ± 5.76		
Cog.I	23.66 ± 5.11		
T-DEP	28.96 ± 7.81		
Anh.T	16.23 ± 4.88		
Dys.T	12.72 ± 3.85		

*SD, standard deviation; n, number of the participants; only child, the only one alive child of his or her parents; left-behind child, not living together with his or her parents before 10 years of age; BIS, barratt impulsiveness scale; Non.I, non-planning impulsiveness of the BIS; Mot.I, motor impulsiveness of the BIS; Cog.I, cognitive impulsiveness of the BIS; T-DEP, trait depression scale; Anh.T, trait anhedonia of the T-DEP; Dys.T, trait dysthymia of the T-DEP.*

### Assessment

#### Trait Depression Scale

The Trait Depression Scale is a subscale of the State Trait Depression Scale (ST-DEP) developed by Charles D. Spielberger in 1995 (11). Unlike the Zung Self-Rating Depression Scale (SDS) (34) and Beck Depression Inventory (BDI) (35), it is used to measure one’s long-term depressive emotion (trait) rather than the state within 1 or 2 weeks. In addition, ST-DEP neglects the items relevant to somatization, retaining the items reflecting cognition and emotion, which are divided into two facets in both the S-DEP and T-DEP, namely, euthymia and dysthymia. Euthymia is the “existence of positive affect,” while dysthymia is the “existence of negative affect” (11). Given that the euthymia subscale is reversely scored, it represents “lack of positive affect,” namely, “anhedonia.” The scale consists of thirty-two items, with sixteen items each in the S-DEP and T-DEP. Of the sixteen items of the T-DEP, eight items represent anhedonia, and the other eight items represent dysthymia. The Chinese version was translated and revised by Lei et al. (36). The reliability and construct validity of both the S-DEP and T-DEP were demonstrated among samples from college students (36). In this study, the T-DEP was shown to be reliable, with McDonald’s omega values of 0.910 (95% CI = [0.892, 0.928]) and 0.861 (95% CI = [0.831, 0.892]) in anhedonia and dysthymia subscales, respectively.

#### Barratt Impulsiveness Scale

The Barratt Impulsiveness Scale was originally developed by Barratt (37). It has been widely used to evaluate impulsive traits and behavioral patterns of healthy individuals or those who have impulse control disorder, borderline personality disorder, or other relevant mental disorders (9). There are thirty items in BIS, which are divided into three subscales, namely, “non-planning,” “motor,” and “cognitive” impulsiveness, representing “lack of forethought,” “acting without inhibition,” and “acting without thinking,” respectively. Li and Philips translated the 11th version of the BIS into Chinese (Barratt Impulsiveness Scale 11th Chinese version, BIS-11-CV) (38), and they retained six items, revised five items, and replaced nineteen items. The reliability and validity of the BIS-11-CV have been shown to be good among Chinese samples from communities and colleges (27). However, according to Li and Philips’ study (38), Item 13 (one item for “non-planning” impulsiveness) is more likely a feature of “cognitive” impulsiveness, while Item 24 (one item for “cognitive” impulsiveness) is more likely a feature of “motor” impulsiveness. We accepted the conclusion in this study; thus, the BIS-11-CV had good reliability with a coefficient of internal consistency in the present sample. The McDonald’s omega values of the three subscales were 0.872 (95% CI = [0.844, 0.900]), 0.836 (95% CI = [0.806, 0.867]), and 0.846 (95% CI = [0.812, 0.881]).

### Statistical Analyses

#### Descriptive Statistics

Demographic information and descriptive statistics were analyzed using IBM SPSS Statistics version 20.0 (39).

#### Network Analyses

RStudio version 1.4 with R 4.0.4 was used to conduct the network analyses (40).

##### Network Estimation

Gaussian graphical model (GGM) (41) is the basic method of cross-sectional network analysis. Based on GGM, the partial correlation model (PCM) (41) can eliminate spurious correlation. This study used the Graphic Least Absolute Shrinkage and Selection Operator (GLASSO) algorithm (42) to estimate the partial correlations of each observed variable, which could shrink the weak correlations to zero within the network to obtain a more stable network. The GLASSO algorithm was applied with the EBICglasso function of the R “qgraph” package (43). The network was visualized as nodes and edges of different colors and thicknesses. The red edges represent negative partial correlations, while the blue edges represent positive partial correlations. Thicker and darker edges represent stronger strength of correlations.

##### Centrality

Centrality represents the numbers, strength, and closeness of the correlations of one node with others in a network. The basic indexes of centrality are degree, closeness, and betweenness (43). Closeness and betweenness include “short path length, SPL” (28), considering all the direct and indirect correlations of one node with others, which allows the importance of the node to be evaluated. In the weighted network, the sum of weights of all edges of one node represents the centrality index, which is named “strength.” Studies have shown the strength centrality to be more stable than closeness and betweenness (44, 45). However, for a network with both positive and negative edges, a previous study has shown that “expected influence,” which is the sum of the value of all edges connecting to one node, is more appropriate (46). In the present study, the expected influence was chosen to represent the centrality index.

##### Stability and Accuracy Analyses

These two indexes were calculated by the R “bootnet” package (47). Because centrality statistics can be unreliable, accuracy and stability analyses were necessary. Therefore, the *post-hoc* stability and accuracy analyses were conducted. The 95% confidence intervals (95% CIs) were calculated for the accuracy of edge weights with bootstrapping. The narrower the 95% CI was, the more accurate the edge weights were. The recommended value of edge weight accuracy is not less than 0.5 (47). The stability of centrality indexes was estimated by calculating the correlation stability (CS) coefficient with case-dropping bootstrapping. The CS coefficient should not be lower than 0.25 and better be more than 0.5 (47).

##### Bridge

The Bridge indexes are usually used to describe overlapping nodes in studies on mental disorders (48). The bridge expected influence can indicate the risk of contagion among different disorders (29). In this study, the bridge expected influence was applied to illustrate the overlap of the two traits to prove that those who were vulnerable to depression had certain characteristics of impulsiveness. It was calculated by the R package “networktools” (49). The higher the bridge expected influence was, the greater the overlap was.

##### Network Comparison

To examine the effects of gender (male and female), “only child” (only child and non-only child), and “left-behind child” (left-behind child and non-left-behind child) on the network, we conducted network comparisons by the R “NetworkComparisonTest” package, applying permutation test to compare the network invariance (the network structure pattern) and the global strength invariance (the sum of the weight of the edges within the network) (30). The network comparisons for the three factors were performed, respectively.

## Results

### Demographic Information and Descriptive Statistics

The demographic information and descriptive statistics are shown in Table 1.

### Network Structure

**FIGURE 2:**
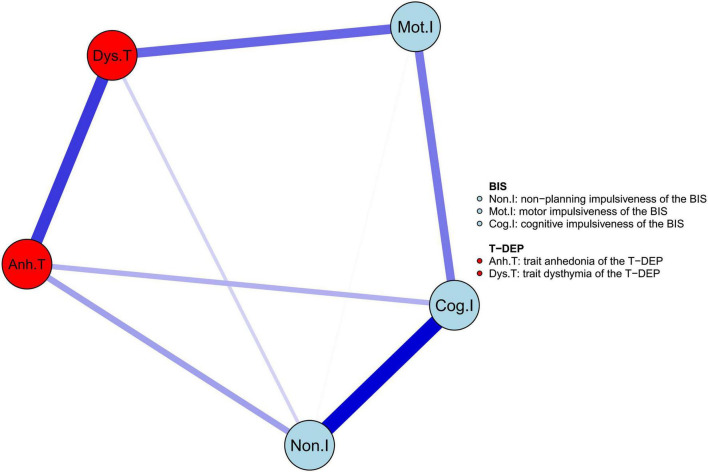
The network structure of trait depression and impulsiveness in the youth. The edge weight values are listed in Supplementary Table 2.

The network of trait depression and impulsiveness is shown in Figure 2. Eight of ten possible connections were not zero. Within trait depression, “trait anhedonia” and “trait dysthymia” were closely connected, with an edge weight of 0.400. Within impulsiveness, “cognitive” impulsiveness was linked with “non-planning” and “motor” impulsiveness with the edge weights of 0.515 and 0.271, respectively, while “non-planning” and “motor” impulsiveness were connected, with an edge weight of 0.006. Between the two traits, “trait anhedonia” was related to “non-planning” impulsiveness (weight = 0.193) and “cognitive” impulsiveness (weight = 0.157), and “trait dysthymia” was connected with “motor” impulsiveness (weight = 0.309) and “non-planning” impulsiveness (weight = 0.089). Among these, the weights of the edges “Mot.I–Dys.T” and “Non.I–Dys.T” were neither significantly different (95% CI_bootstrap_ = [–0.002, 0.459]) nor were the weights of the edges “Non.I–Anh.T” and “Cog.I–Anh.T” (95% CI_bootstrap_ = [–0.301, 0.246]) (refer to Table 2). Meanwhile, the weight of the edge “Non.I–Cog.I” was significantly larger than those of “Mot.I–Cog.I” (95% CI_bootstrap_ = [0.045, 0.454]) and “Non.I–Mot.I” (95% CI_bootstrap_ = [0.258, 0.687]) (refer to Table 2). The weights of the edges “Cog.I–Dys.T” and “Mot.I–Anh.T” were shrunk to zero after applying the GLASSO algorithm, which indicated that these two linkages were of the least importance in this network.

**TABLE 2:** Edge weight comparisons.

Edge 1	Edge 2	95% CI_bootstrap_ of Δ weight (edge 1–edge 2)	
		Lower	Upper
Mot.I–Dys.T	Non.I–Dys.T	–0.002	0.459
Cog.I–Anh.T	Non.I–Anh.T	–0.301	0.246
Non.I–Cog.I	Mot.I–Cog.I	0.045	0.454
Mot.I–Cog.I	Non.I–Mot.I	–0.036	0.457
Non.I–Cog.I	Non.I–Mot.I	0.258	0.687

*Non.I, non-planning impulsiveness of the BIS; Mot.I, motor impulsiveness of the BIS; Cog.I, cognitive impulsiveness of the BIS; Anh.T, trait anhedonia of the T-DEP; Dys.T, trait dysthymia of the T-DEP; 95% CI_bootstrap_, 95% confidence interval computed with the bootstrapping method. The level of significance test was alpha = 0.05 (corrected by Bonferroni correction).*

### Centrality

**FIGURE 3:**
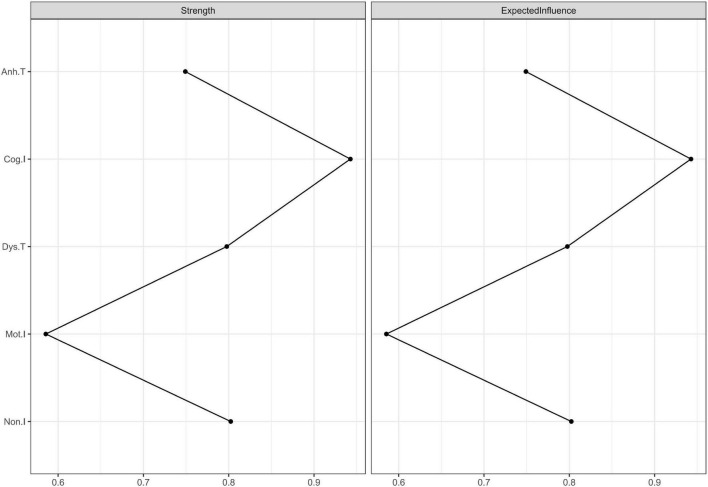
Centrality plots of strength and expected influence. Because there was no negative edge weight in this network, the values of strength and expected influence were the same. The strength and expected influence values are listed in Supplementary Table 3.

We used expected influence as the index of centrality. As shown in Figure 3, “Cog.I” had the highest expected influence values (the expected influence values are listed in Table 3), indicating that this node was the most important one in the network and had the strongest connections to other nodes. “Mot.I” had the lowest expected influence value, indicating that this node was the least important one in the network. However, the significance test showed that only the comparison between “Cog.I” and “Mot.I” was statistically significant (refer to Table 3).

**TABLE 3:** Expected influence comparisons of each node.

Node 1	Node 2	95% CI_bootstrap_ of Δ expected influence (node 1–node 2)	
		Lower	Upper
Cog.I	Mot.I	0.083	0.667
Non.I	Cog.I	–0.503	0.228
Dys.T	Cog.I	–0.451	0.151
Anh.T	Cog.I	–0.556	0.094
Non.I	Mot.I	–0.032	0.507
Dys.T	Mot.I	–0.011	0.520
Anh.T	Mot.I	–0.081	0.436
Dys.T	Non.I	–0.278	0.251
Anh.T	Non.I	–0.436	0.202
Anh.T	Dys.T	–0.413	0.224

*Non.I, non-planning impulsiveness of the BIS; Mot.I, motor impulsiveness of the BIS; Cog.I, cognitive impulsiveness of the BIS; Anh.T, trait anhedonia of the T-DEP; Dys.T, trait dysthymia of the T-DEP; 95% CI_bootstrap_, 95% confidence interval computed with the bootstrapping method. The level of significance test was alpha = 0.05 (corrected by Bonferroni correction).*

### Stability and Accuracy

The number of bootstrapping samples was 2,000 when calculating both the edge weight accuracy and the CS coefficient of expected influence. In this network, the edge weight accuracy was 0.75 (the bootstrap mean of the edge weight is plotted in Supplementary Figure 1), higher than the recommended 0.5 (47). The CS coefficient of expected influence was 0.44 (the average correlation with the original sample for expected influence is plotted in Supplementary Figure 2), higher than the recommended 0.25 (47). These results indicate that the centrality statistics were stable and accurate.

### Bridge

**FIGURE 4:**
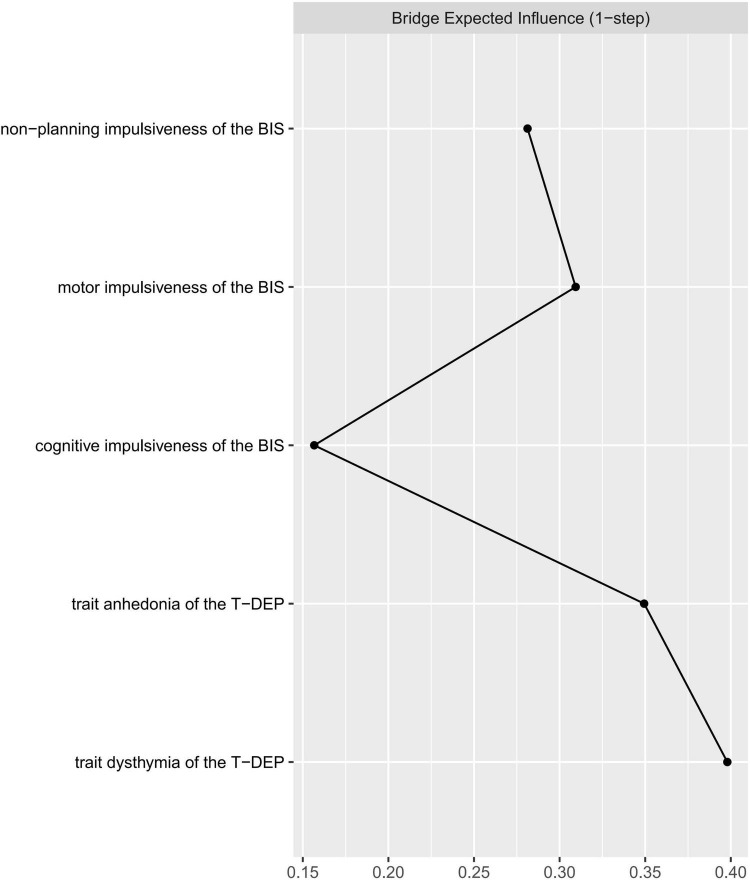
Centrality plot of the bridge expected influence between trait depression and impulsiveness.

The bridge index is usually used in symptom networks to determine which symptoms have the greatest risk of contagion between two symptom groups. However, for personality networks, it can be applied to describe which trait components linked the different personalities closest. We regarded impulsiveness and trait depression as two communities when calculating the bridge expected influence values. The trait components were represented by facets (five facets in total). According to Figure 4, the most important bridge trait component was “trait dysthymia,” and the bridge expected influence value of which was 0.398. However, only the difference between “trait dysthymia” and “cognitive” impulsiveness was significant (95% CI_bootstrap_ = [0.011, 0.461]) (refer to Table 4) (the bridge expected influence values are listed in Supplementary Table 4). The CS coefficient of bridge expected influence was calculated with bootstrapping (*n* = 2,000), resulting in a value of 0.29 (the average correlation with the original sample for bridge expected influence is plotted in Supplementary Figure 3), which was higher than the recommended 0.25 (47). This indicates that the bridge centrality statistics were stable.

**TABLE 4:** Bridge expected influence comparisons of each node.

Node 1	Node 2	95% CI_bootstrap_ of Δ bridge expected influence (node 1–node 2)	
		Lower	Upper
Cog.I	Mot.I	–0.347	0.172
Non.I	Cog.I	–0.168	0.377
Dys.T	Cog.I	0.011	0.461
Anh.T	Cog.I	–0.094	0.380
Non.I	Mot.I	–0.269	0.253
Dys.T	Mot.I	–0.072	0.336
Anh.T	Mot.I	–0.162	0.262
Dys.T	Non.I	–0.077	0.345
Anh.T	Non.I	–0.199	0.263
Anh.T	Dys.T	–0.425	0.200

*Non.I, non-planning impulsiveness of the BIS; Mot.I, motor impulsiveness of the BIS; Cog.I, cognitive impulsiveness of the BIS; Anh.T, trait anhedonia of the T-DEP; Dys.T, trait dysthymia of the T-DEP; 95% CI_bootstrap_, 95% confidence interval computed with the bootstrapping method. The level of significance test was alpha = 0.05 (corrected by Bonferroni correction).*

### Network Comparisons

We did not find differences between male and female in network invariance (M = 0.163, *p* = 0.614) or global strength (S = 0.238, male = 1.972, female = 2.211, *p* = 0.199). This indicates that gender does not affect the network structure pattern or connection strength. There were also no significant differences between the “left-behind child” group and the “non-left-behind child” group in network invariance (M = 0.233, *p* = 0.312) or global strength (S = 0.156, “left-behind child” = 1.827, “non-left-behind child” = 1.982, *p* = 0.438). However, although there was no significant difference between “only child” group and “non-only child” group in network invariance (M = 0.179, *p* = 0.487), we found a difference in global strength invariance (S = 0.408, “only child” = 1.889, “non-only child” = 2.297, *p* = 0.038). This result indicates that the “non-only child” group has a stronger global connection than the “only child” group among impulsiveness and trait depression facets.

## Discussion

In the present study, we adopted the network analysis method to explore the associations between different facets of trait depression (e.g., “trait anhedonia” and “trait dysthymia”) and impulsiveness (e.g., “non-planning,” “motor,” and “cognitive” impulsiveness) and their relative importance. We demonstrate that “trait anhedonia” is connected with “non-planning” and “cognitive” impulsiveness but not with “motor” impulsiveness and that “trait dysthymia” is connected with “non-planning” and “motor” impulsiveness but not with “cognitive” impulsiveness. In addition, according to the expected influence, “cognitive” impulsiveness is the most important facet in the network, which can link other facets globally. Meanwhile, “trait dysthymia” is the most important facet linking trait depression with impulsiveness. Among the demographic variables, “only child” affects the network global strength, while “gender” and “left-behind child” do not, which indicates that the facets of trait depression and impulsiveness are more closely connected with each other in “non-only child” than in “only child.”

### Network Structure

As demonstrated in our hypothesis, what we mainly care about are the associations of the three facets of impulsiveness with “trait anhedonia” and “trait dysthymia.” There are two disagreements between the results and the hypothesis. First, “cognitive” impulsiveness is connected with “trait anhedonia” rather than “trait dysthymia.” The cognitive component of impulsiveness mentioned earlier is measured by a behavioral task, which mainly refers to error-related brain activities. However, in this study, “cognitive” impulsiveness is measured by a self-report questionnaire, which represents a more general impulsive cognitive process (not thinking thoroughly before action). In addition, the correlation between anhedonia and the cognitive component of impulsiveness is confirmed in a study, in which “cognitive” is directly measured by brain functional imaging (50). Second, “non-planning” impulsiveness is connected not only with “trait anhedonia” but also with “trait dysthymia.” There may be two possible explanations for this result. One is that both “trait anhedonia” and “trait dysthymia” have unmotivating components, which can be observed from the item meanings of T-DEP (36). Another is that “non-planning” impulsiveness includes both unmotivating components that are related to “trait anhedonia” and other components that may be related to “trait dysthymia.” The result that “non-planning” impulsiveness is connected with “trait anhedonia” agrees with the result of a previous study, considering the non-planning reward process and anhedonia symptoms (50). However, the relationship between “trait dysthymia” and the “non-planning” impulsiveness needs further consideration in the future.

The connection between “trait dysthymia” and “motor” impulsiveness confirms the hypothesis. The positive linkage between “motor” impulsiveness and “trait dysthymia” agrees with the result of a previous study, showing that dysthymic symptoms are related to cognitive control evaluated by behavioral measurement and ERN (26) because behavioral measuring of cognitive control includes behavioral inhibition component that is the feature of “motor” impulsiveness. The connections between “trait dysthymia” and “non-planning” impulsiveness and between “trait dysthymia” and “motor” impulsiveness are not significantly different. However, the edge weight of “trait dysthymia—motor impulsiveness” has a trend to be larger (refer to Table 2). These findings indicate that “trait dysthymia” tends to be mainly linked with “motor” impulsiveness.

In conclusion, we tend to believe that “non-planning” and “cognitive” impulsiveness are the trait features of anhedonia and “motor” impulsiveness is the trait feature of dysthymia.

In addition, within trait depression, “trait anhedonia” and “trait dysthymia” are closely linked with each other. This indicates that trait depression seems to be an integral construct, which agrees with the description in DSM-5 (51) that anhedonia and dysthymia are the two cardinal symptoms of major depressive disorder. However, within impulsiveness, the association between “non-planning” and “cognitive” impulsiveness is stronger compared with the other two associations. This may be because “non-planning” and “cognitive” impulsiveness are mainly cognitive features, while “motor” impulsiveness is mainly a behavioral feature (9). Therefore, impulsiveness seems not to be a simple structure.

### Expected Influence

The expected influence reveals the importance of each trait facet (46). The five trait facets are included in a single structure, which may be regarded as the vulnerable personality for depressive disorder. “Cognitive” impulsiveness is a relatively more important facet in this study. This indicates that it is connected with other facets more widely or more closely. “Cognitive” impulsiveness represents quick thinking without inhibition (38), which is a stable feature of cognitive processing. The results infer that the cognitive feature of impulsiveness may be the core factor. It agrees with the cognitive hypothesis of depression (52). “Motor” impulsiveness is the least important facet in this structure. This indicates that “motor” impulsiveness only composes a small part of the vulnerability to depression. One possible reason is that “motor” impulsiveness includes fewer cognitive components than the other two impulsiveness facets (38). However, the cognitive component is an important factor in depression (52). Another possible reason is that “motor” impulsiveness, defined as impaired behavioral inhibition (9), is included in the behavioral inhibition system that has a weak correlation with anhedonia (53). The other three facets (e.g., “non-planning” impulsiveness, “trait anhedonia,” and “trait dysthymia”) have no significant difference in relative importance from neither “cognitive” impulsiveness nor “motor” impulsiveness. This result reveals that “cognitive” impulsiveness only has a significantly larger weight than “motor” impulsiveness, which indicates that none of the five facets is statistically dominant in this network. Nevertheless, “cognitive” impulsiveness has the trend to be dominant.

### Bridge Expected Influence

Regarding trait depression and impulsiveness as two different systems, we use the bridge index (48) to find the common components between them. “Trait dysthymia” is a common component that links trait depression with impulsiveness. A possible explanation is that “trait dysthymia,” as a habitual mood distress feature, could be affected by the inability of controlling temper and behavior (9). “Cognitive” impulsiveness has the lowest bridge expected influence, despite its highest expected influence. This indicates that it less directly links with “trait dysthymia” and “trait anhedonia” but through other trait facets. The bridge expected influence of the other three facets (e.g., “trait anhedonia,” “motor” impulsiveness, and “non-planning” impulsiveness) has no significant difference from neither “cognitive” impulsiveness nor “trait dysthymia,” which indicates that none of the five facets is statistically more important than others in linking impulsiveness with trait depression. However, “trait dysthymia” has the trend to be the most important facet bridging the two traits.

### Network Comparisons

The results of the network comparisons show that gender, “only child,” and “left-behind child” does not affect the network structure. Previous studies have revealed that gender (54), “only child” (55), and “left-behind child” (56) are the factors that affect the prevalence of depressive symptoms. However, the present study mainly focuses on the inner correlation pattern. This may lead to the non-significant effects of demographic variables on network structure. Global strength represents the degree of associations among the facets of trait depression and impulsiveness. In previous studies, gender’s effects on the association between impulsiveness and depressive symptoms are inconsistent. Some of them report significant effects (57, 58) but others do not (59, 60). In the present study, gender does not affect global strength, which agrees with the non-significant results of the previous studies. Further studies are needed to figure out whether gender’s non-significant effect on the global strength of the associations among the facets of impulsiveness and depression is stable across symptom-level and trait-level. To the best of our knowledge, although there are studies exploring the effect of “only child” and “left-behind child” on depression (55, 56), few consider their influences on the association between impulsiveness and depression. Both “only child” and “left-behind child” include the factor of childhood experience, which is the basis of personality formation and development (61, 62). In our study, “only child” does influence the global strength of this personality network but “left-behind child” does not. This may be due to the psychosocial confounders (e.g., parenting pattern and family structure), which we have not taken into consideration.

### Implications

Above all, the results indicate that “cognitive” impulsiveness is an underlying feature, while “trait dysthymia” is a key feature that links impulsiveness with trait depression. For the prevention of depression, it seems that “cognitive” impulsiveness needs more consideration because it widely influences the whole vulnerability network. “Trait dysthymia” needs more attention when considering the reciprocal effects of impulsiveness and trait depression. A better intervention of “trait dysthymia” may reduce the likelihood of the co-existence of trait depression and impulsiveness, which can decrease the risk of the aftermath led by depression and impulsiveness together (e.g., suicide). When considering the subtypes of depression, dysthymia needs more attention on both “trait dysthymia” and “motor” impulsiveness, while anhedonia needs more attention on “trait anhedonia,” “cognitive,” and “non-planning” impulsiveness. In addition, “non-only children” need more attention in the prevention of depression, because they are more possible to have both impulsiveness and trait depression than “only children.”

### Limitations

There are some limitations to this study. First, the sample comprised college students and residents in Chongqing, China. It is unknown whether the results can be expanded to a wider sample. Second, the data were collected in a cross-sectional manner before morbidity, which was not sufficient to determine whether the network is on trait level. Third, some psychosocial confounders were not taken into consideration, which might affect the network structure and strength. In the future, depressive populations in the premorbid, state, and remitted stages can all be recruited. The relations existing across the three stages would be stronger evidence for the trait hypothesis. More participants need to be recruited from different areas and ethnicities. More psychosocial factors that may influence personality can be included to reduce bias.

## Conclusion

The present study confirms the correlation between trait depression and impulsiveness personality, finding that “trait anhedonia” is associated with “non-planning” and “cognitive” impulsiveness, while “trait dysthymia” is associated with “motor” impulsiveness. Therefore, in the prevention of depression, different aspects of impulsiveness should be considered respectively regarding anhedonia and dysthymia. In addition, “cognitive” impulsiveness is an underlying feature of the vulnerability to depression, and “trait dysthymia” is a key factor linking impulsiveness with trait depression. Therefore, “cognitive” impulsiveness and “trait dysthymia” are critical to the prevention of depression.

## Data Availability Statement

The raw data supporting the conclusions of this article will be made available by the authors, without undue reservation.

## Ethics Statement

The studies involving human participants were reviewed and approved by Medical Ethics Committee of Army Medical University. Written informed consent to participate in this study was provided by the participants’ legal guardian/next of kin.

## Author Contributions

JZ contributed to the design of the research, conducting a questionnaire survey and statistical analyses, and drafting the manuscript. KL contributed to the questionnaire survey, writing the R script, and revising the manuscript. YX contributed to the questionnaire survey, arranging the raw data, and performing statistical analyses. ZF contributed to the design of the whole study and the critical revision of the manuscript. All authors have read and revised the final manuscript.

## Conflict of Interest

The authors declare that the research was conducted in the absence of any commercial or financial relationships that could be construed as a potential conflict of interest.

## Publisher’s Note

All claims expressed in this article are solely those of the authors and do not necessarily represent those of their affiliated organizations, or those of the publisher, the editors and the reviewers. Any product that may be evaluated in this article, or claim that may be made by its manufacturer, is not guaranteed or endorsed by the publisher.

## Abbreviations

BIS: Barratt impulsiveness scale
BIS-11-CV: Barratt impulsiveness scale 11th Chinese version
Non.I: non-planning impulsiveness of the BIS
Mot.I: motor impulsiveness of the BIS
Cog.I: cognitive impulsiveness of the BIS
ST-DEP: state trait depression scale
T-DEP: trait depression scale
Dys.T: trait dysthymia of the T-DEP
Anh.T: trait anhedonia of the T-DEP
SDS: self-rating depression scale
BDI: beck depression inventory
GGM: Gaussian graphical model
GLASSO: graphic least absolute shrinkage and selection operator
PCM: partial correlation model
SPL: short path length
CI: confidence interval
CS: correlation stability
SD: standard deviation
PRISMA: preferred reporting items for systematic reviews and meta-analyses.
