# Supplementary material for: Network Analysis of the Relationship Between Trait Depression and Impulsiveness Among Youth
Source: Front Psychiatry. 2022 Jun 17;13:916332. doi: 10.3389/fpsyt.2022.916332 (PMC9247242; doi:10.3389/fpsyt.2022.916332)
Supplement: Supplementary file 1 [file Data_Sheet_1.doc]

**Table1 The definitions of the facets of different impulsiveness models**

| model | facet | definition |
| --- | --- | --- |
| Fineberg’s model(1) | motor impulsiveness | inability to stop motor responses |
|  | disadvantageous decision-making | difficulty weighting options and taking appropriate risks |
|  | choice impulsiveness | excessive discounting of delayed reinforcement |
|  | reflection impulsiveness | reduced tendency to collect salient information before decision-making |
| Barratt’s model(2) | motor | impaired behavioral inhibition |
|  | non-planning | impulsive decision regardless of delayed rewards/lack of forethought |
|  | cognitive | acting without sufficient thinking or information processing |

**Table 2** Edge weight values of the network

| edge | edge weight value |
| --- | --- |
| Non.I--Cog.I | 0.515 |
| Anh.T--Dys.T | 0.400 |
| Mot.I--Dys.T | 0.309 |
| Mot.I--Cog.I | 0.271 |
| Non.I--Anh.T | 0.193 |
| Cog.I--Anh.T | 0.157 |
| Non.I--Dys.T | 0.089 |
| Non.I--Mot.I | 0.006 |
| Cog.I--Dys.T | 0 |
| Mot.I--Anh.T | 0 |

Note: Non.I – non-planning impulsiveness of the BIS; Mot.I – motor impulsiveness of the BIS; Cog.I – cognitive impulsiveness of the BIS; Anh.T – trait anhedonia of the T-DEP; Dys.T – trait dysthymia of the T-DEP.

**Table 3 Strength and expected influence values of each node**

| node | strength | expected influence |
| --- | --- | --- |
| Non.I | 0.802 | 0.802 |
| Mot.I | 0.586 | 0.586 |
| Cog.I | 0.943 | 0.943 |
| Anh.T | 0.749 | 0.749 |
| Dys.T | 0.798 | 0.798 |

Note: Non.I – non-planning impulsiveness of the BIS; Mot.I – motor impulsiveness of the BIS; Cog.I – cognitive impulsiveness of the BIS; Anh.T – trait anhedonia of the T-DEP; Dys.T – trait dysthymia of the T-DEP.

Table 4 Bridge expected influence values of each node

| node | bridge expected influence |
| --- | --- |
| Non.I | 0.281 |
| Mot.I | 0.309 |
| Cog.I | 0.157 |
| Anh.T | 0.349 |
| Dys.T | 0.398 |

Note: Non.I – non-planning impulsiveness of the BIS; Mot.I – motor impulsiveness of the BIS; Cog.I – cognitive impulsiveness of the BIS; Anh.T – trait anhedonia of the T-DEP; Dys.T – trait dysthymia of the T-DEP.


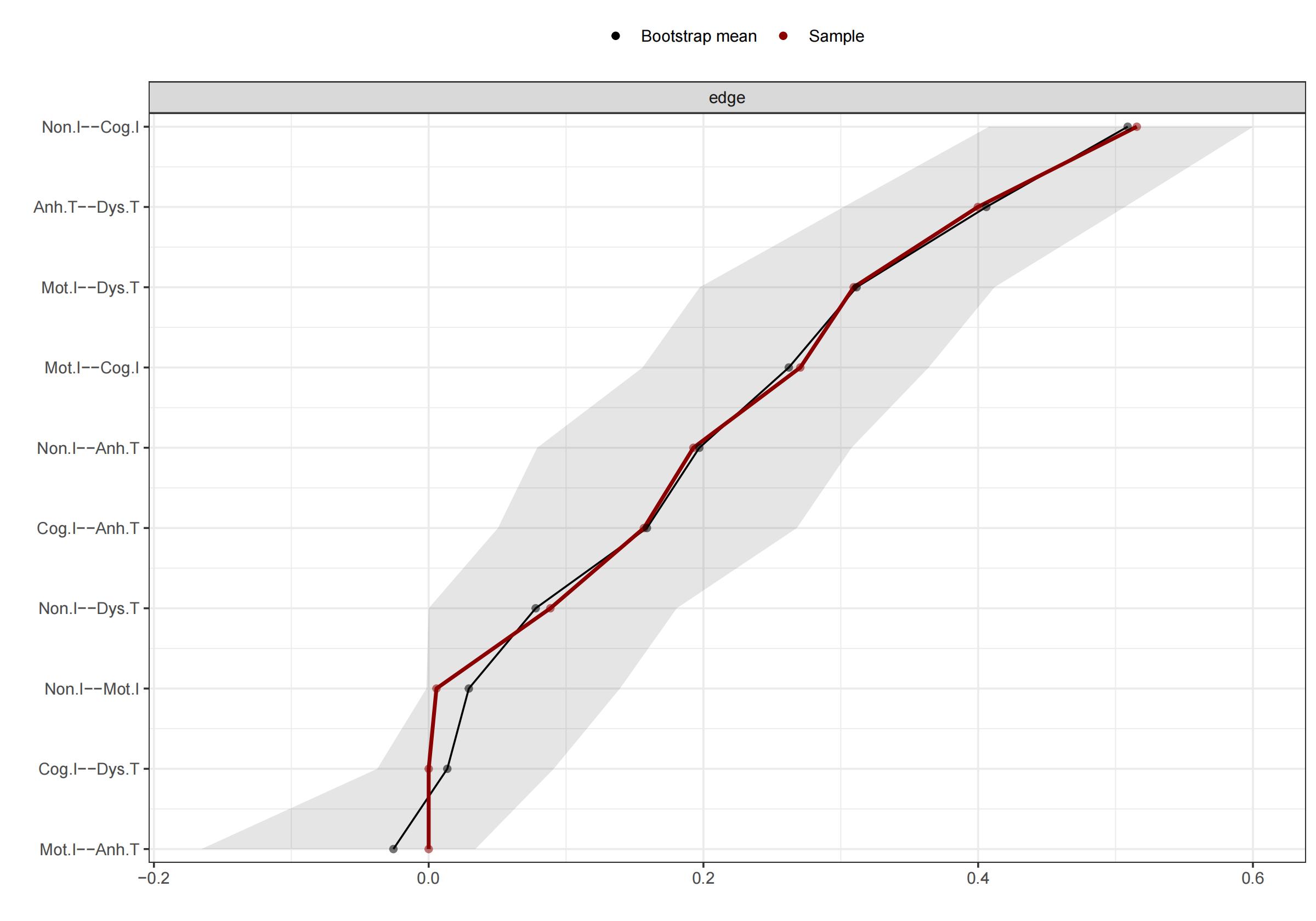


**Figure 1 The bootstrap mean of the edge weight**


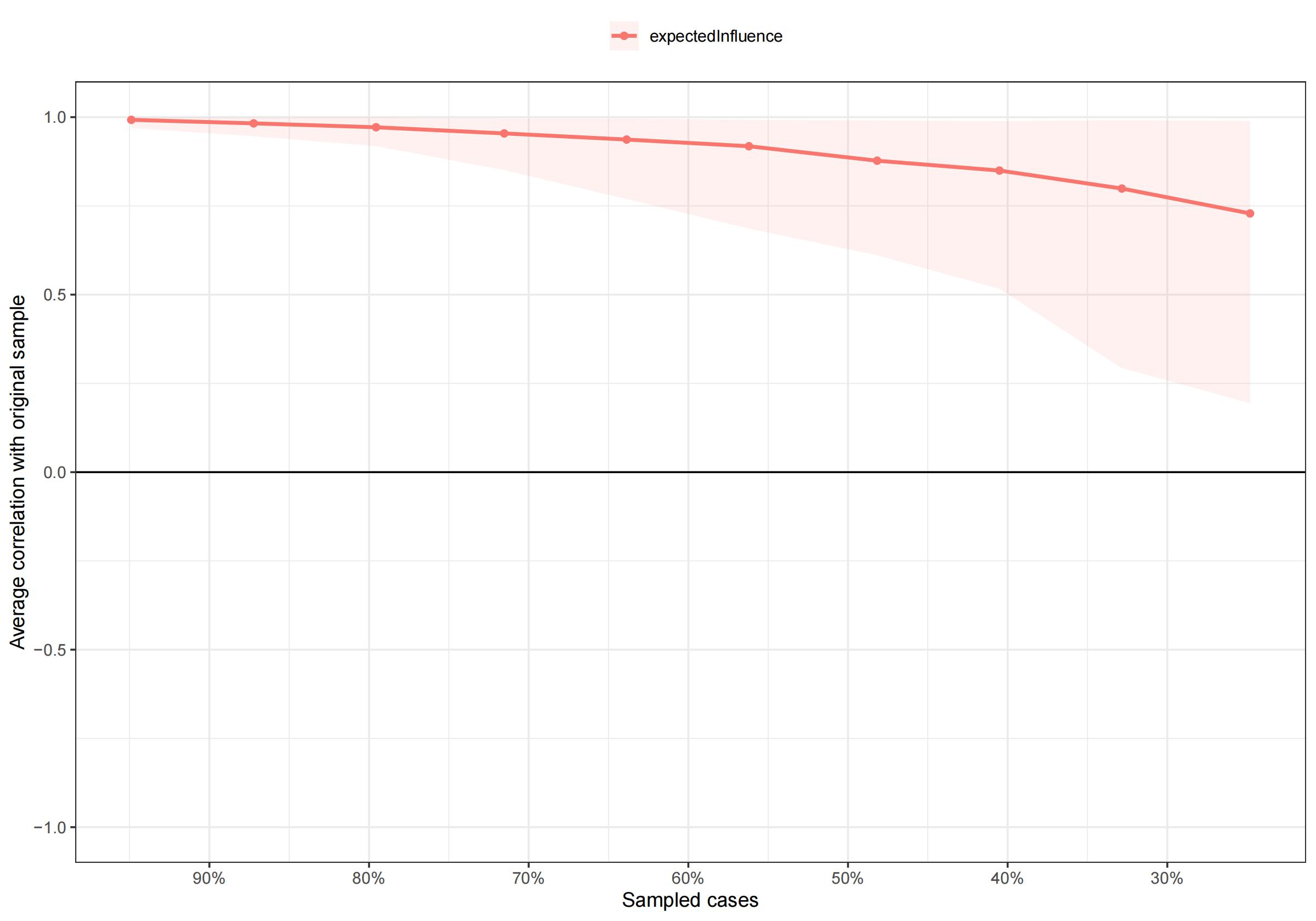


**Figure 2 The average correlation with the original sample for expected influence**


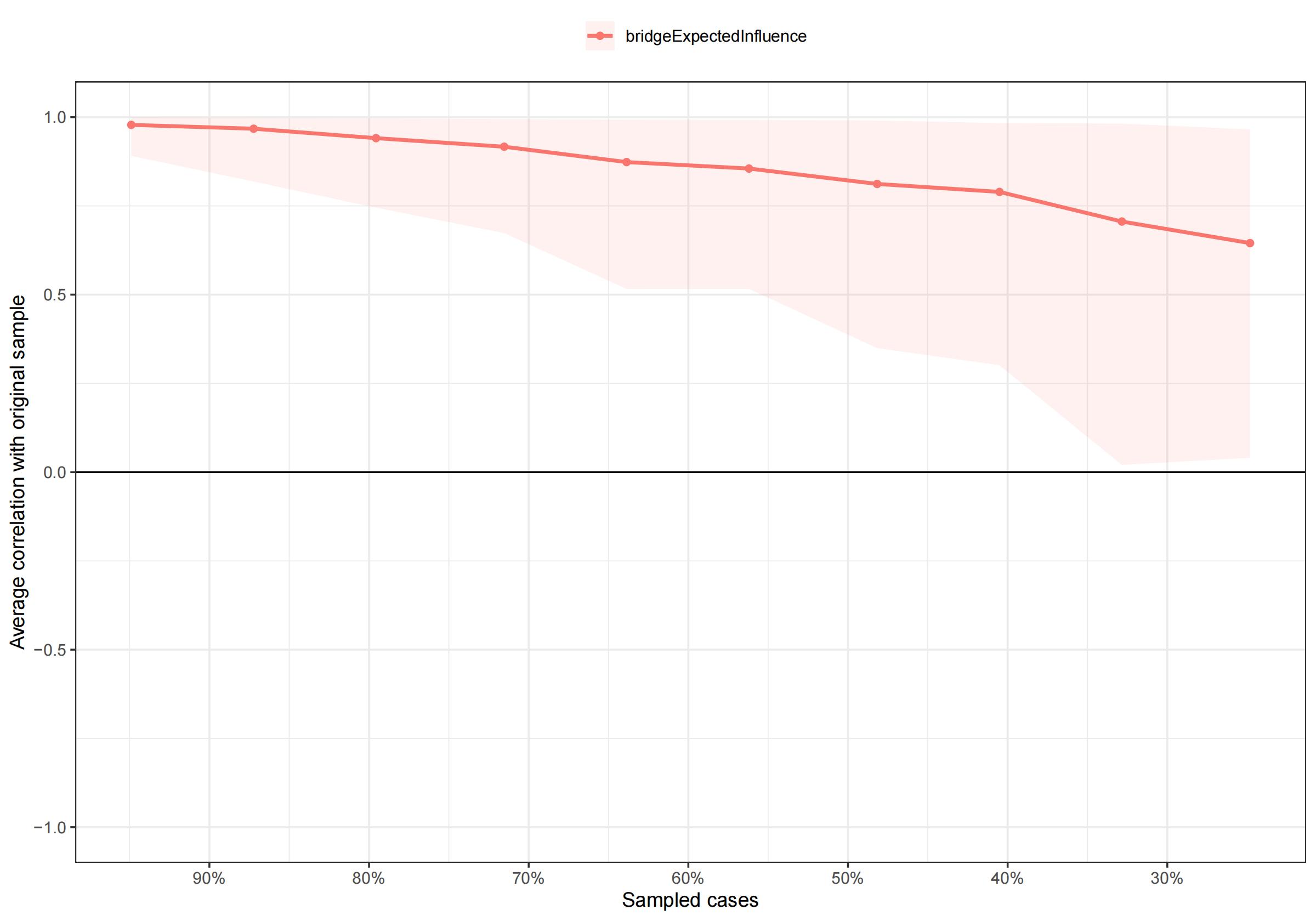


**Figure 3 The average correlation with the original sample for bridge expected influence**

**Reference**

1. Fineberg NA, Chamberlain SR, Goudriaan AE, Stein DJ, Vanderschuren LJ, Gillan CM, et al. New developments in human neurocognition: clinical, genetic, and brain imaging correlates of impulsivity and compulsivity. CNS Spectr. 2014;19(1):69-89.

2. Stanford MS, Mathias CW, Dougherty DM, Lake SL, Anderson NE, Patton JH. Fifty years of the Barratt Impulsiveness Scale: An update and review. Personality & Individual Differences. 2009;47(5):385-95.
